# Structure-guided development of Pb^2+^-binding DNA aptamers

**DOI:** 10.1038/s41598-021-04243-2

**Published:** 2022-01-10

**Authors:** Hehua Liu, Yanqing Gao, Johnsi Mathivanan, Fusheng Shen, Xi Chen, Yangyang Li, Zhiwei Shao, Yixi Zhang, Qiyuan Shao, Jia Sheng, Jianhua Gan

**Affiliations:** 1 Shanghai Public Health Clinical Center, State Key Laboratory of Genetic Engineering, Collaborative Innovation Center of Genetics and Development, School of Life Sciences, Fudan University, 2005 Songhu Road, Yangpu District, Shanghai, 200438 People’s Republic of China; 2 Department of Chemistry, and The RNA Institute, University at Albany, State University of New York, 1400 Washington Avenue, New York, 12222 USA

**Keywords:** X-ray crystallography, Analytical biochemistry, DNA, Biophysical chemistry

## Abstract

Owing to its great threat to human health and environment, Pb^2+^ pollution has been recognized as a major public problem by the World Health Organization (WHO). Many DNA aptamers have been utilized in the development of Pb^2+^-detection sensors, but the underlying mechanisms remain elusive. Here, we report three Pb^2+^-complexed structures of the thrombin binding aptamer (TBA). These high-resolution crystal structures showed that TBA forms intramolecular G-quadruplex and Pb^2+^ is bound by the two G-tetrads in the center. Compared to K^+^-stabilized G-quadruplexes, the coordinating distance between Pb^2+^ and the G-tetrads are much shorter. The T3T4 and T12T13 linkers play important roles in dimerization and crystallization of TBA, but they are changeable for Pb^2+^-binding. In combination with mutagenesis and CD spectra, the G8C mutant structure unraveled that the T7G8T9 linker of TBA is also variable. In addition to expansion of the Pb^2+^-binding aptamer sequences, our study also set up one great example for quick and rational development of other aptamers with similar or optimized binding activity.

## Introduction

Lead (Pb) is one of the most toxic heavy metals known to date. Through mimicking of essential elements such as iron, zinc and calcium, Pb can enter and accumulate in the human body^1^, impairing the functions of multiple organs including kidney, heart and brain^2^. The direct relationships between Pb poisoning and growth^3^, delayed puberty^4^ and hematological changes^5^ have been well documented. Human can be exposed to Pb under diverse circumstances such as battery^6,7^ and automotive manufacturing^8^, refining and smelting^9^. Contaminated drinking water is another important source of Pb exposure^10^ and has occurred in many areas^11,12^.

Owing to its great threat to human health, Pb poisoning has been recognized as one major public problem. To detect the Pb present in human body as well as in the surrounding environment, various methods have been developed. Many methods rely on the traditional analytical techniques such as atomic absorption spectroscopy, anodic stripping voltammetry and reverse-phase high-performance liquid chromatography. Although these methods are sensitive, they require sophisticated equipments. The requirement of complicated sample treatment is another major limitation of these methods. To develop faster and simpler methods for Pb detection, scientists have performed extensive trials and found that some DNA sequences possess high Pb ion (Pb^2+^) binding affinity and specificity. These sequences were termed as Pb^2+^-binding DNA aptamers and have been extensively utilized in Pb^2+^ detector development. Compared to the traditional Pb^2+^ detecting methods, these aptamer-based methods are easier in handling and less expensive.

To date, various Pb^2+^-binding DNA aptamers have been identified^13^. Exampled by PS2.M^14^, PW17, T30695 and the thrombin binding aptamer (TBA), many Pb^2+^-binding DNA aptamers are rich in guanines. Guanine-rich sequences are often observed at the ends of chromosomal telomeres or at the promoter regions of human genes^15^. Interestingly, these sequences are also present in the genomes of many viruses such as Papilloma Virus and Human Immunodeficiency Virus (HIV), playing important regulatory roles in viral replication and gene expression. Guanine-rich sequences are capable of forming quadruplexes^16^. Quadruplexes are normally stabilized by the stacking of multiple Hoogsteen hydrogen-bonded G-tetrads and the electrostatic interactions between the guanines and the cations (commonly K^+^ or Na^+^) residing in the center of the tetrads.

Circular dichroism (CD), UV and/or NMR spectra indicated that Pb^2+^ can cause large conformational changes and induce the quadruplex formation of many DNA aptamers such as PS2.M, PW17 and T30695^13,17^. Compared to K^+^ and Na^+^, Pb^2+^ is more efficient in quadruplex formation of these aptamers. Although the structural basis is not very clear, Pb^2+^-binding aptamers have been utilized in the design of Pb^2+^-controlled DNA gate^13^ and other nanodevices. Assisted by other technologies, such as Fluorescence Anisotropy (FA)^18^, Fluorescent Metal Nanocluster (NCs)^19^ and Forster Resonance Energy Transfer (FRET)^20^, these Pb^2+^-binding aptamers have also been used to develop ultrasensitive Pb^2+^ sensors. Due to their intrinsic difficulty in crystallization, the Pb^2+^-DNA aptamer complex structures are barely available, which hindered our understanding on Pb^2+^-binding mechanisms by DNA aptamers. Here, we report three complex structures of Pb^2+^-TBA, which revealed the detailed basis for Pb^2+^ binding by TBA. In combination with site-specific mutations and CD analyses, our studies revealed the detailed mechanistic insights into the Pb^2+^-TBA interactions and also shed great light for the development of novel Pb^2+^-binding aptamers.

## Results

### Overall structure of TBA-Pb^2+^ complex

**Figure 1:**
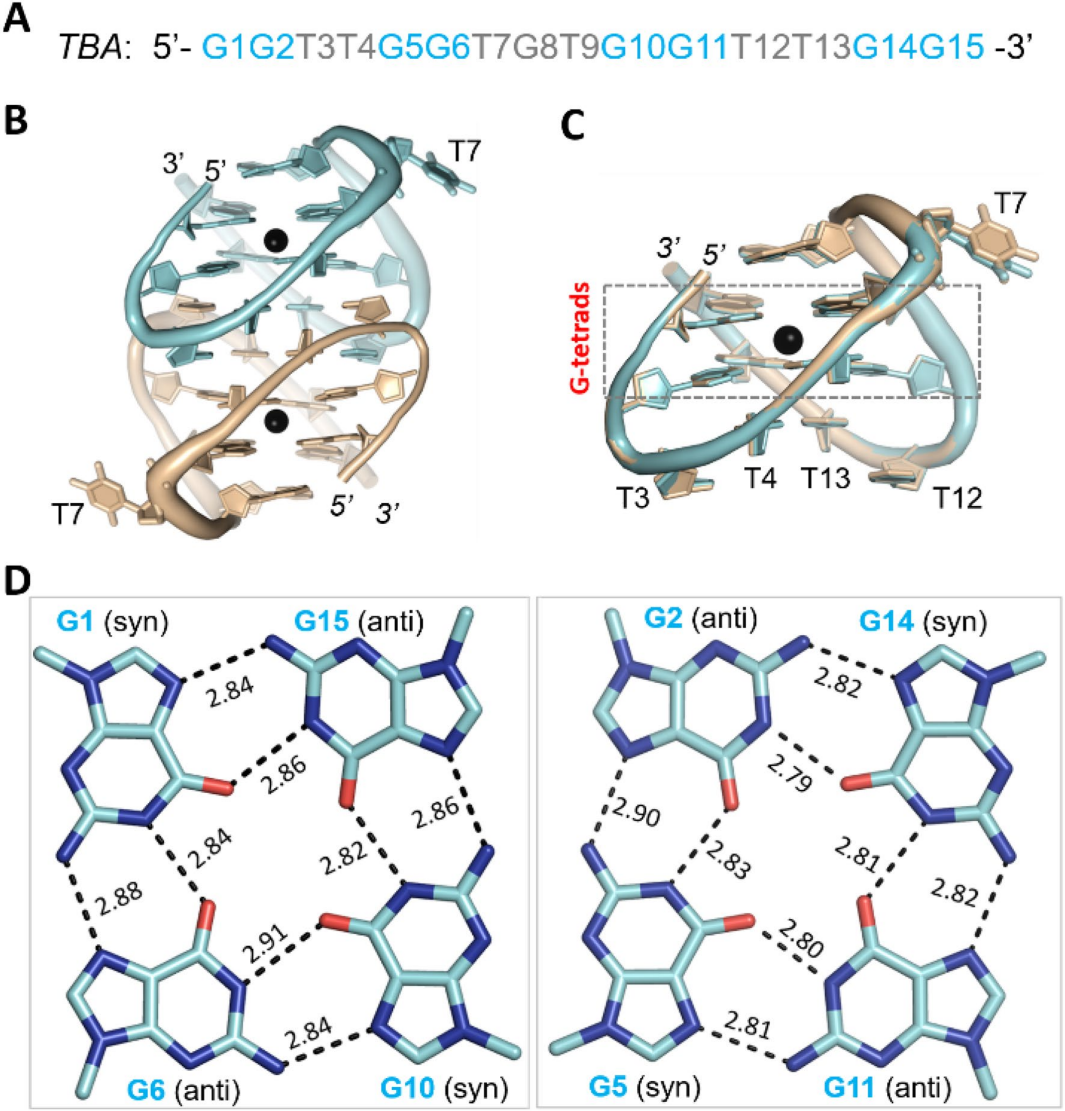
TBA-Pb^2+^ complex structure. (**A**) Sequence of TBA aptamer. (**B**) Overall folding of the A-form TBA-Pb^2+^ complex structure. (**C**) Superposition of the two TBA-Pb^2+^ complexes. (**D**) The G-tetrads observed in the A-form TBA-Pb^2+^ complex structure. The detailed Hoogsteen H-bond distances are labelled with numbers (Å). In (**B,C**), TBA molecules A and B are shown as cartoons in cyan and wheat, respectively. Pb^2+^ ions are shown as black spheres. In (**D**), the C-atoms are colored in cyan for the G-tetrads of TBA molecule A, respectively. Figures were displayed using PyMOL (http://www.pymol.org/).

To reveal the underlying basis of Pb^2+^-binding by DNA aptamers, we performed extensive crystallization trials. Although no crystals were obtained for PS2.M, PW17 or T30695, we solved two TBA-Pb^2+^ complex structures with high-resolution in both A-form and B-form (Table 1). As calculated by the Matthews Coefficient program, the solvent content (44.7%) of the A-form structure is higher than that (40.0%) of the B-form structure. The A-form structure belongs to the tetragonal space group P4_1_2_1_2; it contains two TBA-Pb^2+^ complexes per asymmetric unit (Fig. 1B). The B-form structure belongs to the orthorhombic space group P2_1_2_1_2_1_; per asymmetric unit contains six TBA-Pb^2+^ complexes. Due to their different space groups, packing of the TBA-Pb^2+^ complexes are very different in the A- and B-form structures (Fig. S1A,B). However, the overall folding of the eight TBA-Pb^2+^ complexes are similar. The root mean square deviations value (RMSD) between the two TBA-Pb^2+^ complexes is only 0.15 Å within the A-form structure; and, it ranges from 0.3 to 0.5 Å between the complexes of the A- and B-form structures.

**Table 1:** Data collection and refinement statistics.

Structure (PDB ID)	TBA-Pb^2+^ complex (A-form) 7D31	TBA-Pb^2+^ complex (B-form) 7D32	G8C-Pb^2+^ complex 7D33
**Data collection**^a^			
Space group	P4_1_2_1_2	P2_1_2_1_2_1_	C2
Cell parameter			
a (Å)	40.0	27.3	51.4
b (Å)	40.0	53.1	78.0
c (Å)	99.9	152.5	101.7
α (°)	90.0	90.0	90.0
β (°)	90.0	90.0	113.6
ρ (°)	90.0	90.0	90.0
Wavelength (Å)	0.9793	0.9793	0.9793
Resolution (Å)	30.0–1.4	30.0–1.7	37.4–2.1
Last shell (Å)	1.45–1.4	1.76–1.70	2.23–2.1
Completeness (%)	97.9 (89.6)	96.0 (84.4)	96.5 (98.5)
Redundancy	14.4 (3.8)	5.7( 2.6)	6.7 (6.7)
I/σ (I)	44.9 (3.3)	9.6 (1.9)	6.7 (2.4)
Rmerge (%)	10.9 (38.1)	14.6 (43.2)	22.5 (93.9)
**Refinement**			
Resolution (Å)	27.2–1.4	26.4–1.7	25.3–2.1
R_work_ (%)/R_free_ (%)	12.6/14.8	18.9/21.5	21.2/24.0
No. of atoms			
DNA	630	1890	1560
Pb^2+^	2	6	5
Water	57	85	70
R.m.s. deviations			
Bond length (Å)	0.007	0.006	0.005
Bond angle (°)	0.997	0.918	0.751

^a^Values in parentheses are for the last resolution shell.

Compared to the B-form structure, the resolution (1.4 Å) of the A-form TBA-Pb^2+^ complex structure is higher, therefore it was used for further structural analysis. TBA is composed of 15 nucleotides. As depicted in Fig. 1C, each TBA molecule folds into an antiparallel chair-like quadruplex, which can be divided into four regions: two TT linkers (T3T4 and T12T13), one TGT linker (T7G8T9) and the central G-tetrads. The central G-tetrads are formed by eight G nucleotides, in which G1, G6, G10 and G15 arranged in one layer and G2, G5, G11 and G14 arranged in a second layer (Fig. S2). In both layers, two G nucleotides adopt *syn* conformation and the other two adopt *anti* conformation. Interestingly, although the conformations of the G nucleotides varied within the two G-tetrads, the interactions mediated by the individual G nucleotide conserved. As depicted in Fig. 1D, each G nucleotide forms four Hoogsteen hydrogen bond interactions. The average in-plane N1–O6 and N2–N7 distances are 2.83 Å and 2.85 Å, respectively. Compared to other G-quadruplex structures, both the N1-O6 and N2-N7 distances are shorter in the TBA-Pb^2+^ complex structure (Table 2). The two G-tetrads of TBA-Pb^2+^ complex form stable hydrophobic stacking interactions, the average between-plane O6–O6 distance is only 2.98 Å, which is significantly shorter than other quadruplexes.

**Table 2:** Mean distances (Å) observed in the A-form TBA-Pb^2+^ complex and reported quadruplex structures.

	A-form TBA-Pb^2+^(PDB:7D31)	thrombin-TBA complex (PDB: 4DII)	oxytricha nova telomeric quadruplex (PDB: 3NZ7)
Pb^2+^–O6	2.65		
K^+^–O6		2.82	2.86
O6–O6 (between G-tetrads)	2.98	3.31	3.44
O6–O6 (within G-tetrads)	3.12	3.25	3.28
N1–O6 H-bond	2.83	2.83	2.88
N2–N7 H-bond	2.85	2.89	2.92

### Pb^2+^ forms tight coordination with TBA

**Figure 2:**
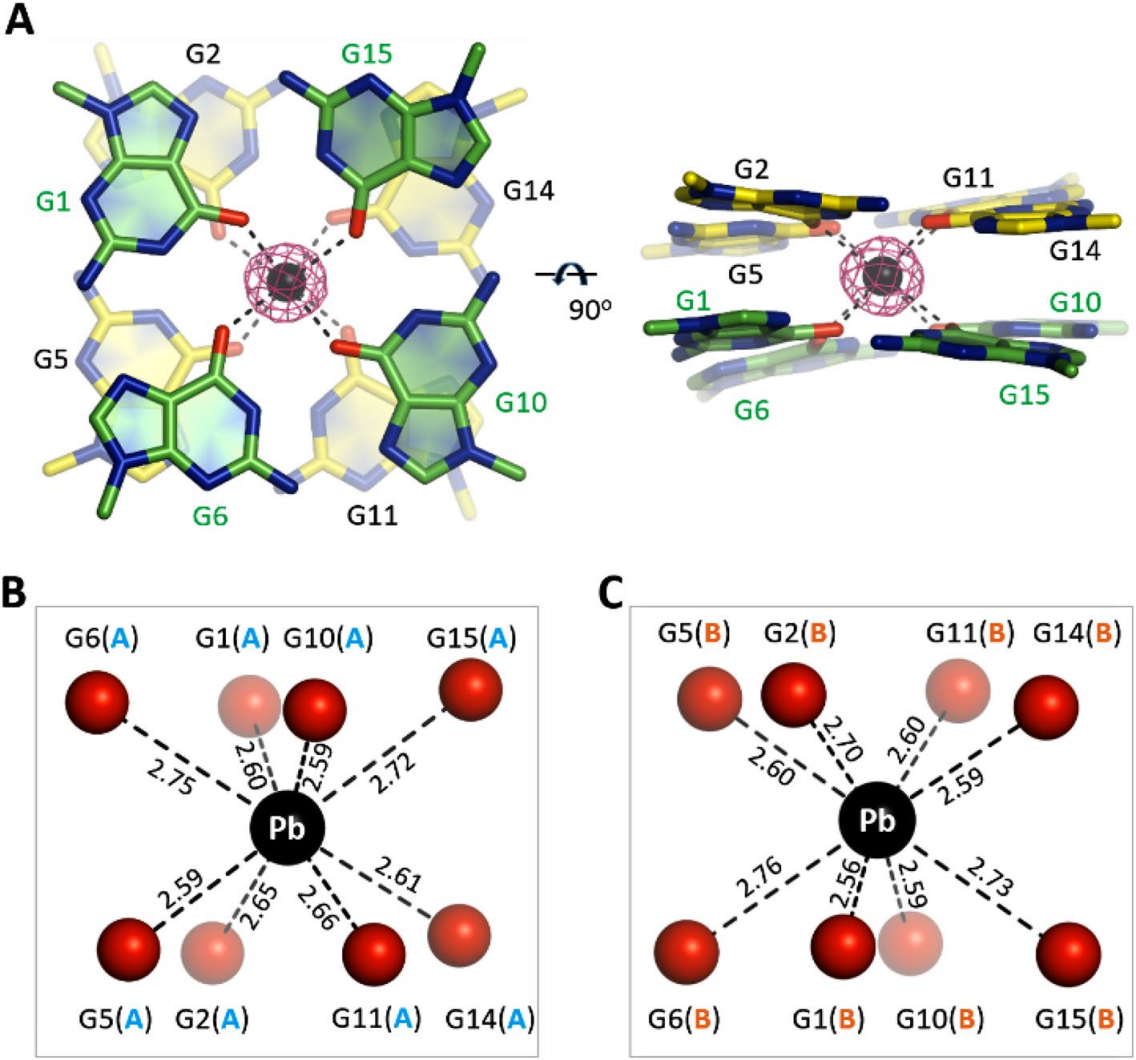
Pb^2+^ coordination by TBA. (**A**) Electron density and coordination of the Pb^2+^ ions. The two G-tetrads are shown as sticks. Pb^2+^ ions are shown as black spheres. The 2F_o_ − F_c_ electron density maps of Pb^2+^ are contoured at 10 σ level. (**B,C**) The detailed coordinating geometry and coordinating distances (Å) of the Pb^2+^ ions. Pb^2+^ and the O6 atoms of the G-tetrads are shown as spheres in black and red, respectively. Figures were displayed using PyMOL (http://www.pymol.org/).

TBA is one of the earliest identified Pb^2+^-binding aptamers. Although it is not preferred by many other quadruplexes, Pb^2+^ is effective in folding and stabilizing the structures of TBA. In the A-form TBA-Pb^2+^ complex structure, each TBA molecule captures one Pb^2+^ ion. The Pb^2+^ ion resides in-between the two G-tetrads (Fig. 2A), coordinating with the eight O6 atoms of the G-tetrads (Fig. 2B,C). The shortest coordination (2.56 Å) forms between Pb^2+^ and the G1 nucleotide of TBA molecule B. In addition, Pb^2+^ forms several short coordination (~ 2.60 Å) with other G nucleotides. The average Pb^2+^–O6 coordinating distance is only 2.65 Å in the A-form TBA-Pb^2+^ complex structure (Table 2). Similar Pb^2+^–O6 coordination and coordinating distance are also observed in the B-form TBA-Pb^2+^ complex structure.

Compared to Pb^2+^, monovalent cations (especially K^+^ and Na^+^) are more common in stabilizing quadruplex structures. Due to its great potential in inhibiting HIV replication, TBA received much more attentions than other DNA aptamers^21–23^. CD and NMR studies confirmed that K^+^ can induce quadruplex formation of TBA, but the quadruplex is very dynamic and requires high concentration of K^+^. We were able to obtain the crystals for the TBA-Pb^2+^ complex. However, in consistent with its intrinsic dynamic, no crystal of TBA-K^+^ complex could be obtained in previous and our studies. Although it is not preferred by other quadruplexes, these observations indicated that Pb^2+^ is more powerful than K^+^ in TBA quadruplex folding and stabilization.

No crystal structure of TBA-K^+^ complex is available to date, but one crystal structure of TBA-thrombin complex (PDB_ID: 4DII)^24^ has been reported, which captured one K^+^ in-between the two G-tetrads. The high-resolution crystal structures of many other K^+^-stabilized quadruplexes have also been reported, including oxytricha nova telomeric quadruplex (PDB_ID: 3NZ7)^25^. To reveal the basis for the strong TBA-stabilizing ability of Pb^2+^, we analyzed the TBA-Pb^2+^ complex, the TBA-thrombin complex and the oxytricha nova telomeric quadruplex. We found that the coordination geometries of Pb^2+^ and K^+^ are actually very similar in the three structures. However, compared to the average Pb^2+^–O6 distance, the average K^+^–O6 coordinating distance is about 0.21 Å longer (Table 2). In addition, the average within-plane and between-plane O6–O6 distances are also longer in the K^+^-stabilized quadruplexes. The ionic radius of Pb^2+^ is 1.29 Å, which is smaller than that (1.51 Å) of K^+^. However, compared to K^+^, Pb^2+^ is more positive in charge. Most likely, the smaller ionic radius and stronger electropositivity of Pb^2+^ play critical roles in the tighter coordination, the more compacted G-tetrad conformations and the higher TBA-Pb^2+^ stability.

### Dimerization is important for TBA-Pb^2+^ crystallization

**Figure 3:**
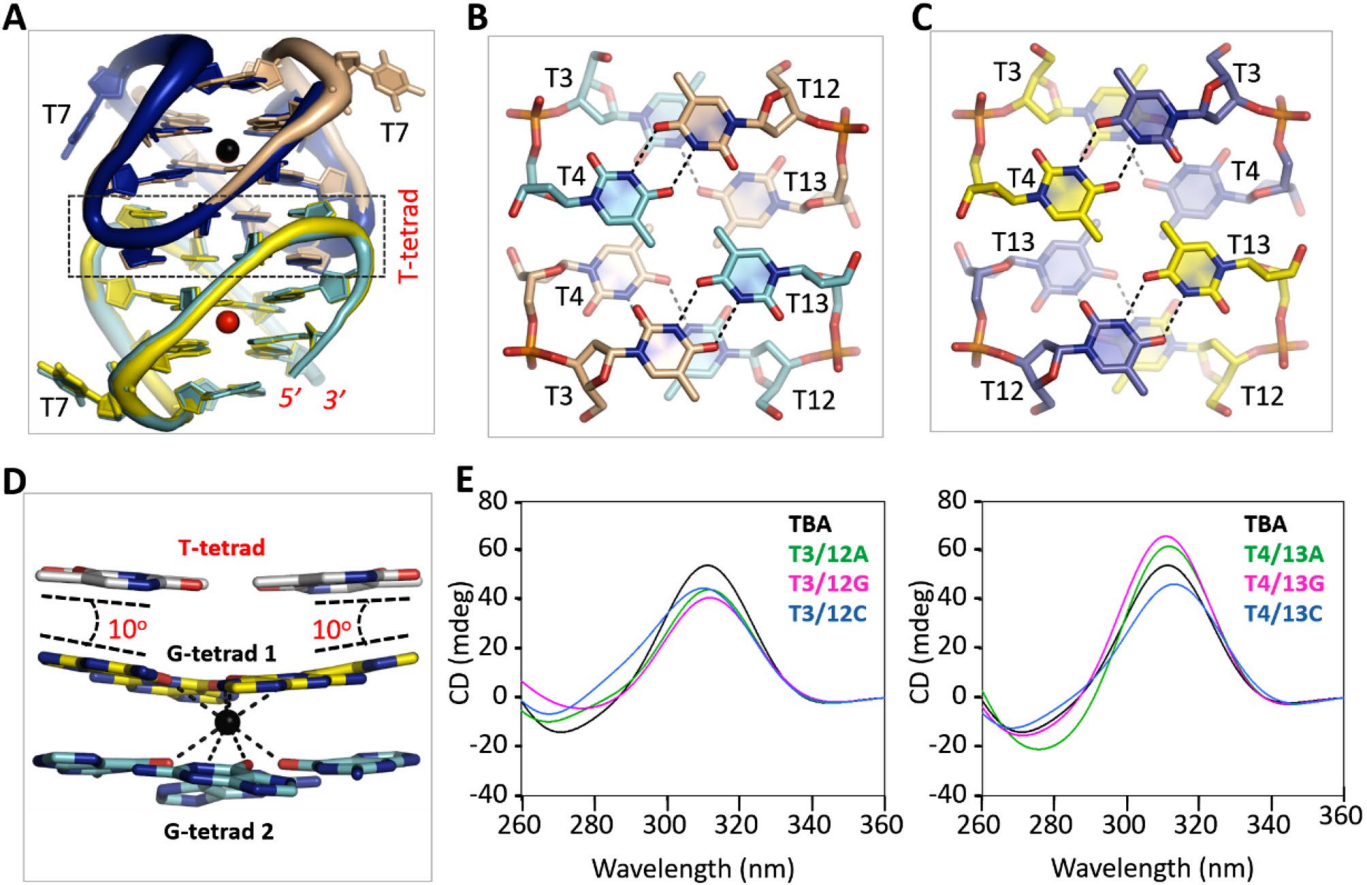
Functional characterization of the TT linkers. (**A**) Structural superposition of the A-form complex with the EF dimer of the B-form complex, which is colored in yellow, blue, and red. The A-form complex is colored as in Fig. 1A. (**B**) Dimerization mediated by the TT linkers of the A-form structure. (**C**) Dimerization mediated by the TT linkers of the EF dimer of the B-form structure. (**D**) Stacking with the pseudo T-tetrad and tilting of the G-tetrad 1. (**E**) CD spectra analysis of the native and TT linker mutated TBAs. Except the mutations listed in the figure, the sequences of the mutants are identical to that of TBA.TBA (native or mutant) and Pb^2+^ concentrations are 10 μM and 50 μM, respectively. Structural figures were displayed using PyMOL (http://www.pymol.org/).

As depicted in Fig. 1B, the two TBA-Pb^2+^ complexes assembled into one dimer in the A-form structure. In fact, the six TBA-Pb^2+^ complexes within the asymmetric unit of the B-form structure also assembled into dimers: dimer AB, CD and EF (Fig. S3A). The overall fold of the AB and CD dimers is very similar to that of the A-form structure (Fig. S3B,C). The overall fold of the EF dimer is also very similar to other dimers, but the relative orientation of the EF dimer is different from others. As depicted in Fig. 3A, when the EF dimer is superimposed with the AB dimer of the A- or B-form structure, there is one 180° rotation (along the central vertical axis) between the F and B molecules.

Dimerization of the TBA-Pb^2+^ complexes is mediated by the two TT linkers. For the AB (or CD) dimers in the A- and B-form structures (Fig. 3B), T3 and T4 arrange in two layers; similar conformations were also adopted by T12 and T13. However, instead of T12 or T13 from the same molecule, T3 and T4 form hydrogen (H) bond interactions with T13 and T12 from the partner molecule, respectively (Fig. S4A,B). T3, T4, T12 and T13 also arrange in two layers in the EF dimer of the B-form structure (Fig. 3C). However, due to the rotation of the F molecule, T3 and T4 of molecule F interact with T4 and T3 of molecule E, respectively. Likewise, T12 and T13 of the two molecules interact with each other (Fig. S4C,D).

To investigate the functional importance of the dimerization mediated by the TT linkers, we designed six TBA mutants with TT linker mutation, including T3/12A, T3/12G, T3/12C, T4/13A, T4/13G, and T4/13C (Table 3). Like native TBA, we performed extensive crystallization trials for all the TT linker mutated sequences. However, none of these sequences could produce crystal. These observations indicated that the TT linkers-mediated dimerization is critical for the crystal growth of TBA-Pb^2+^ complex.

**Table 3:** Sequences of native and mutated TBAs.

Name	Sequence (from 5′ to 3′)
TBA	GG**TT**GG**TGT**GG**TT**GG
T3/12A	GG**A**TGGTGTGG**A**TGG
T3/12G	GG**G**TGGTGTGG**G**TGG
T3/12C	GG**C**TGGTGTGG**C**TGG
T4/13A	GGT**A**GGTGTGGT**A**GG
T4/13G	GGT**G**GGTGTGGT**G**GG
T4/13C	GGT**C**GGTGTGGT**C**GG
T7A	GGTTGG**A**GTGGTTGG
T7G	GGTTGG**G**GTGGTTGG
T7C	GGTTGG**C**GTGGTTGG
G8A	GGTTGGT**A**TGGTTGG
G8T	GGTTGGT**T**TGGTTGG
G8C	GGTTGGT**C**TGGTTGG
T9A	GGTTGGTG**A**GGTTGG
T9G	GGTTGGTG**G**GGTTGG
T9C	GGTTGGTG**C**GGTTGG

Significant letters are given in bold.

### TT linkers are changeable for Pb^2+^-binding by TBA

For all TBA-Pb^2+^ dimers observed in the A- and B-form structures, the eight thymidine residues of the TT linkers formed two pseudo T-tetrads (Fig. 3B,C). Maybe due to the presence of 5-methyl groups at the center, no cation could be bound in-between the two T-tetrads or in-between the T- and G-tetrads (Fig. 3D). The pseudo T-tetrad stacks against G-tetrad 1, the G-tetrad formed by G2, G5, G11 and G14. However, compared to the stacking interactions formed between regular G-tetrads, the stacking interactions between the pseudo T-tetrad and the G-tetrad is weaker, which allows ~ 10° tilt of the nucleobases of the G-tetrad and leads to the tight coordination with Pb^2+^.

The TT linker-mediated dimerization is important for TBA-Pb^2+^ complex crystallization. However, in the TBA-thrombin complex structure, TBA exists as a monomer. Superposition with the TBA-Pb^2+^ complex structures showed that T4 and T12 adopt conserved conformations, but the conformation of T3 and T13 are very different (Fig. S5A). Together with its weaker interaction with the G-tetrad and the variable dimerization manner, we speculated that the pseudo T-tetrads are actually very dynamic and may be not critical for Pb^2+^ binding. To investigate this possibility, we performed CD spectra analysis for the native and mutated TBA sequences (Fig. 3E). Upon addition of Pb^2+^, native TBA showed one strong positive peak near 312 nm, which is the most characteristic peak of Pb^2+^-stabilized quadruplexes. Besides TBA-Pb^2+^, this peak was also observed for PS2.M-Pb^2+^ and PW17-Pb^2+^ complexes previously^12,17^. Like native TBA, all the TBA mutants showed positive peak near 312 nm. Compared to that of native sequence, the heights of the 312-nm peaks are slightly lower for the T3/12A, T3/12G, T3/12C and T4/13C mutants, whereas they are increased for the T4/13A and T4/13G mutants.

### TGT linker helps the stabilization of TBA

**Figure 4:**
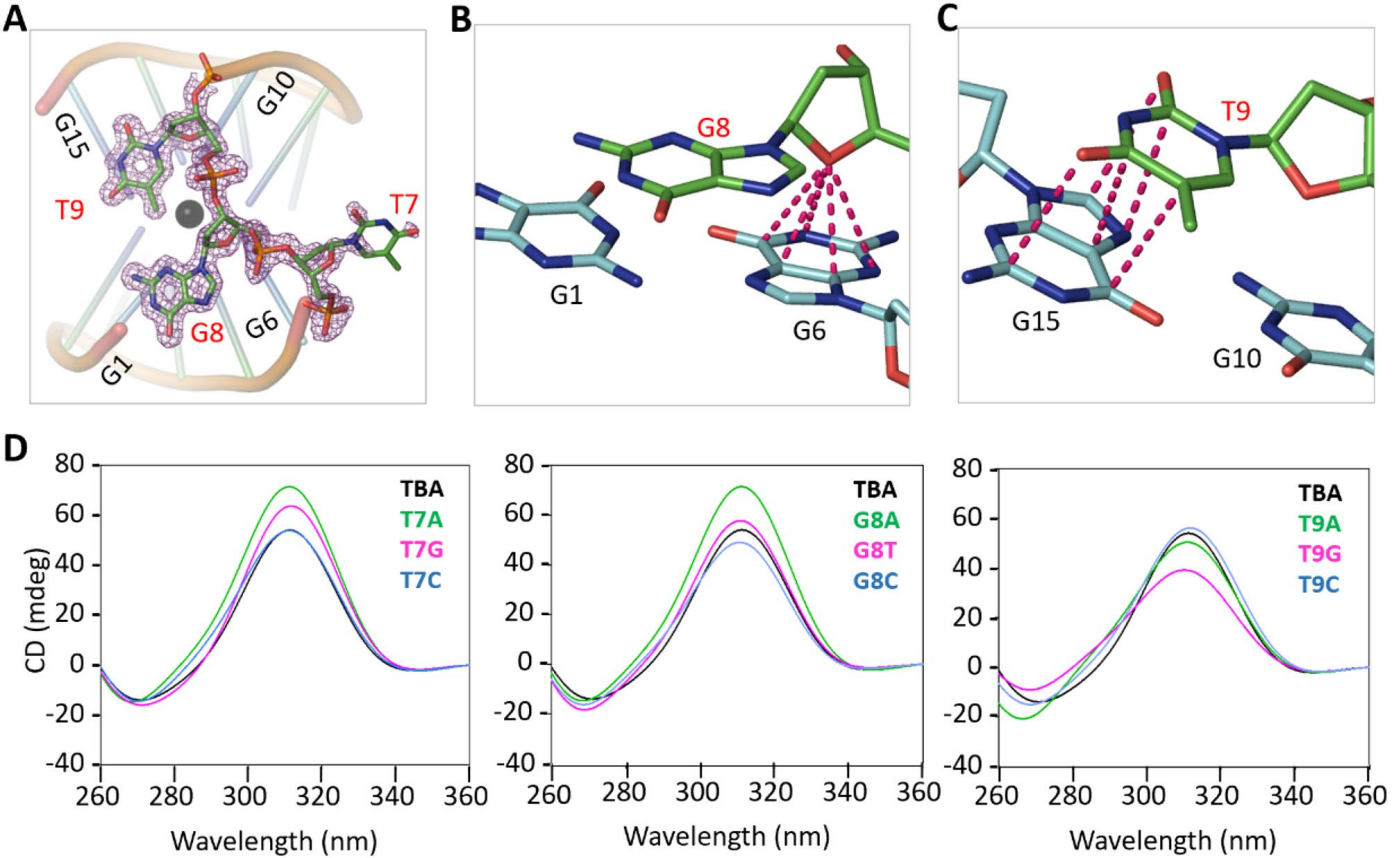
Functional characterization of the TGT linker. (**A**) Detailed conformation of the TGT linker, which are shown as sticks. The 2Fo − Fc electron density maps are contoured at 1.0 σ level. (**B**) O–π interactions formed between the sugar pucker of G8 and the nucleobase of G6. (**C**) Stacking interactions formed between the nucleobases of T9 and G15. (**D**) CD spectra analysis of the native TBA and the TGT linker mutants. Except the mutations listed in the figure, the sequences of the mutants are identical to that of TBA. TBA (native or mutant) and Pb^2+^ concentrations are 10 μM ad 50 μM, respectively. Structural figures were displayed using PyMOL (http://www.pymol.org/).

Unlike the T3T4 and T12T13 linkers, no nucleotide of the T7G8T9 linker forms tetrads in the A- and B-form structures. Instead, the T7G8T9 linker adopts one loop-like conformation (Fig. 4A). The nucleobase of T7 points away from the main body of TBA and does not participate in Pb^2+^ binding. The phosphate backbone of T7 is well organized. However, as indicated by the weak electron densities and high B-factors, the conformation of T7 nucleobase is very flexible. The flexibility of T7 nucleobase could also be further supported by the conformational differences between the A- and B-form structures (Fig. S3B).

G8 and T9 lie on the top of G-tetrad 2, the G-tetrad formed by G1, G6, G10 and G15 (Fig. 4A, Supplementary Fig. S5B). Different from T7, the backbones and nucleobases of G8 and T9 are well defined. The sugar pucker of G8 is perpendicular to the G-tetrad; via its O4′ atom, G8 forms stable O–π interactions with the six-member ring of G6 nucleobase (Fig. 4B). In the A-form structure, the N1 atom of G8 nucleobase forms H-bond interaction with the O5′ atom of G1 (Fig. S6A). Although such interactions were observed for some TBA molecules in the B-form structure, the G8 N1 atoms of other TBA molecules interact with water (Fig. S6B). Similar to G8, the sugar pucker of T9 is also perpendicular to the G-tetrad, but it did not form stable O–π interaction. Instead, T9 forms stacking interaction with G15 (Fig. 4C).

The T7G8T9 linker also adopts loop-like conformation in the TBA-thrombin structure. However, structural superposition showed that the detailed conformations of the T7G8T9 linker is very different from those in the TBA-Pb^2+^ complex (Fig. S5B). May be due to its interactions with symmetry-related thrombin molecules, T9 points away from the main body of TBA in the TBA-thrombin complex. As a compensation, the nucleobase of T7 folds back and forms stacking interactions with G6. Like T7, the nucleobase of G8 also lies on the top of the G-tetrad 2, forming stacking interactions with G10. Although they could be very different in conformations, both TBA-thrombin complex and our TBA-Pb^2+^ complex structures indicated that the T7G8T9 linker interacts with the central G-tetrad and helps the stabilization of TBA.

### TGT linker is variable for Pb^2+^-binding

The structural observations suggested that T7, G8, and T9 might be important for the folding of TBA, whereas they are not directly involved in Pb^2+^-binding. To further clarify the functional roles of the T7G8T9 linker, we synthesized several TBA single-point mutants (Table 3) and performed CD analysis (Fig. 4D). In agree with our structural analysis, substitution of T7 by other nucleotides did not inhibit Pb^2+^-binding by TBA. In fact, the T7A and T7G mutants might be more efficient in Pb^2+^-binding, suggested by the higher 312-nm peaks. Substitution of G8 by C8 (for the G8C mutant) or T8 (for the G8T mutant) has no obvious impact on Pb^2+^-binding; whereas A8 substitution (for the G8A mutant) could lead to stronger Pb^2+^-binding. When T9 was substituted by either A9 or C9 (for the T9A and the T9C mutants), no clear difference on the CD spectra was observed. In contrast to other T7G8T9 linker mutants, the Pb^2+^-binding affinity of T9G mutant is weaker, indicated by the lower peak near 312 nm.

**Figure 5:**
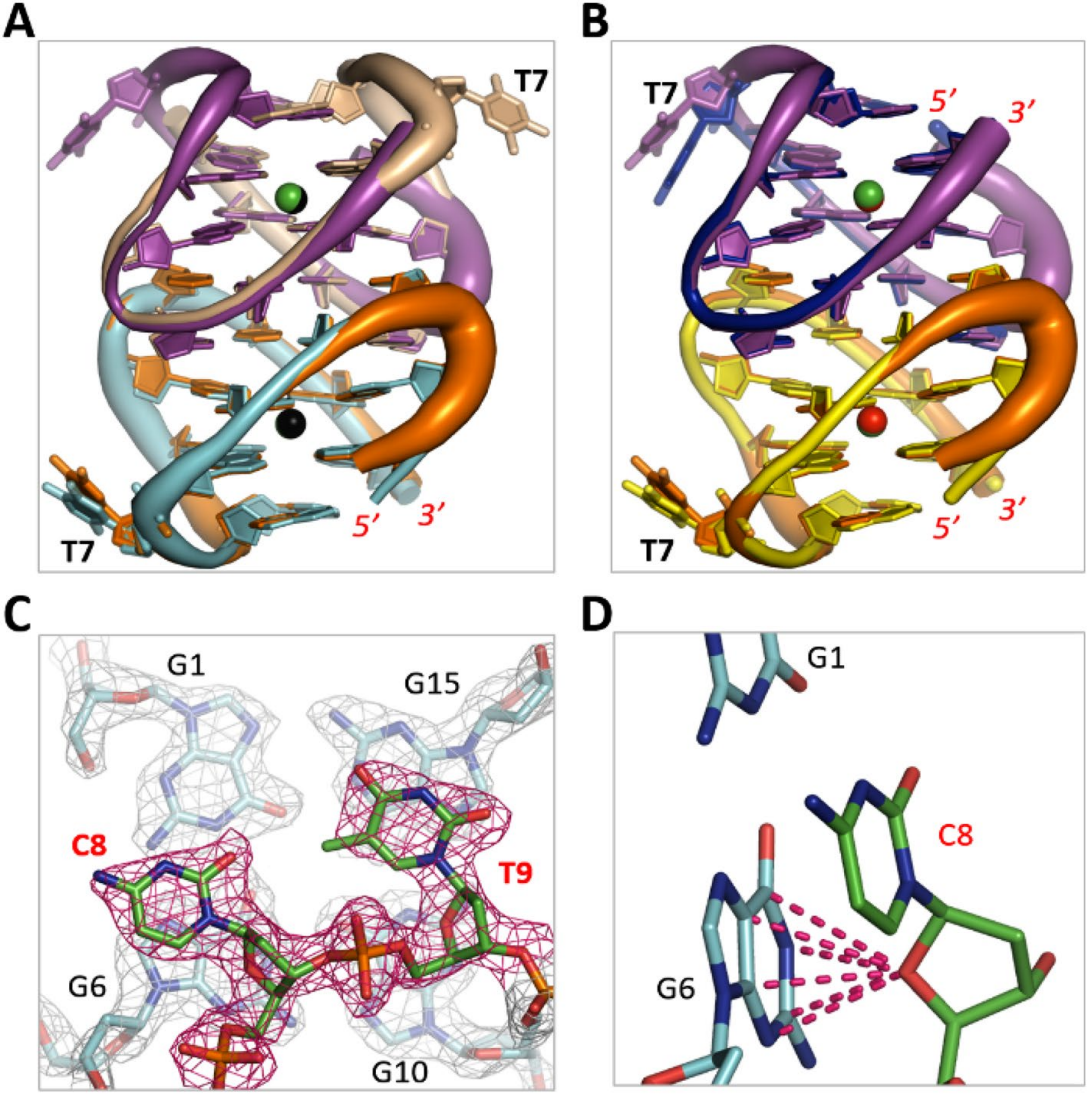
G8C-Pb^2+^ complex structure. (**A**) Superposition of G8C-Pb^2+^ and the A-form TBA-Pb^2+^ structure. (**B**) Superposition of G8C-Pb^2+^ and the EF dimer of the B-form TBA-Pb^2+^ structure. (**C**) Detailed conformation and the 2Fo − Fc electron density map of C8. The maps are contoured at 1.2 σ level. (**D**) O–π interactions formed between the sugar pucker of C8 and the nucleobase of G6. The G8C-Pb^2+^ complexes are colored in orange and magenta in both (**A,B**). Figures were displayed using PyMOL (http://www.pymol.org/).

Similar to native TBA, we also carried out crystallographic studies for the T7G8T9 linker mutants and successfully solved one complex structure of G8C-Pb^2+^ at 2.1 Å resolution (Table 1). The structure belongs to the monoclinic space group C2 and it contains five G8C-Pb^2+^ complexes per asymmetric unit. Four of the complexes formed two intermolecular dimers, whereas the last one formed dimer with symmetric-related molecule. Other than the A-form structure, the overall folding and orientation of the G8C dimers are more similar to dimer EF in the B-form structure (Fig. 5A,B). Due to their different cell units and space groups, packing of G8C-Pb^2+^ complexes is very different from the A-form and B-form structures (Fig. S1C). However, the Pb^2+^-coordinating geometry is almost identical in the three complex structures. C8 is well ordered in the G8C-Pb^2+^ complex structure (Fig. 5C). Similar to G8 in the A- and B-form structures, the sugar pucker of C8 also forms stable O–π interaction with G6 (Fig. 5D), which may lead to the similar CD spectra of G8C mutant and native TBA.

## Discussion

In addition to Pb^2+^-binding DNA aptamers, several DNA enzymes (DNAzymes, such as GR5 and 8-17E) with Pb^2+^ binding ability and specificity have also been discovered^26^. GR5 and 8-17E can cleave RNA in a sequence-specific manner. Owing to their great potential in gene silencing and therapeutics, GR5 and 8-17E have been extensively studied^27,28^. However, due to their intrinsic difficulties in crystallization, the structural information of the Pb^2+^-binding DNAzymes is very limited. The only 8–17 DNAzyme structure was determined by us in 2017, which was facilitated by one protein molecule^29^. As revealed by the crystal structure, Pb^2+^-binding with 8–17 DNAzyme is actually very dynamic, indicated by the relatively low occupancy (~ 50%) of Pb^2+^ in the structure. Besides the enzyme strands, folding of the Pb^2+^-binding DNAzymes also needs RNAs. The RNA strands function as substrates and are degraded during the analysis, which makes the DNAzyme-based Pb^2+^-detection method expensive and difficult in preparation.

Unlike Pb^2+^-binding DNAzymes, the Pb^2+^-binding DNA aptamers are all composed of single stable DNA strands. Compared to the DNAzyme-based methods, the aptamer-based methods are much easier to handle and less expensive. TBA is one of the earliest discovered Pb^2+^-binding DNA aptamers. Like TBA, many other DNA sequences can also assemble into intramolecular quadruplex. Owing to their functional roles in DNA recombination, replication, and transcription, quadruplex structures have attracted significant attentions in the past^30,31^. However, the TBA sequence is very different from the conventional G3-L1-G3-L2-G3-L3-G3 sequence units for identifying potential G-quadruplex structures in bioinformatic studies^32^. Most likely, the unique sequence provides TBA with weak K^+^ binding affinity. The Pb^2+^-binding affinity of TBA is very high and the Pb^2+^-complexed TBA-quadruplex is very stable, which allows us to obtain the crystals and solve three TBA structures at atomic resolution, including two native and one G8C mutant structures. In addition to the detailed coordination, these structures also revealed the detailed basis for the tight Pb^2+^ binding by TBA, such as tilting of the G-tetrad (Fig. 3D).

DNAs are very stable biomolecules. Although not as common as duplexes, DNA quadruplexes also have great potential in nanodevice development. Previous, the Pb^2+^-binding aptamers PS2.M, PW17 and T30695 have been utilized to create K^+^–Pb^2+^-switched DNA gate^13^. The Pb^2+^-stabilized TBA structure is more stable than that stabilized by K^+^. Besides Pb^2+^ binding, TBA can also function as potential inhibitor for HIV replication. Therefore, TBA received much more attentions than other counterpart aptamers^33^. Whereas, the possibility to develop TBA-based nanodevices has not been fully explored. During this study, we noticed that the 5′- and 3′-OH groups are not involved in Pb^2+^-binding by TBA (Fig. S6). Theoretically, florescent groups or additional DNA sequences could be attached to the 5′- or 3′-end of TBA, which will enable the development of novel types of Pb^2+^-sensors or DNA nanodevices.

Guided by the structural observations, we did systematic mutation. CD spectra analysis confirmed that all the TT linker or the TGT linker mutants could bind with Pb^2+^. Compared to native TBA, some mutants might have higher Pb^2+^-binding abilities, such as T4/13A, T4/13G, T7A, T7G and G8A mutants. These results significantly expanded the sequence for the Pb^2+^-binding aptamers. Like Pb^2+^-binding aptamers, many other aptamers have also been reported. Aptamer identification usually involves multiple cycles of in vitro selection and DNA sequencing, which are costy and time-consuming. Our study suggested that structure-based design may be an ideal alternative method to resolve these problems and contribute to the development of new aptamers.

## Methods

### Chemicals

All chemicals and buffers used are of analytical reagent grade. Sodium chloride, lead chloride, lead nitrate, Tris acetate, and acetic acid were purchased from Sigma-Aldrich. Spermine tetra-HCl, sodium cacodylate, (±)-2-Methyl-2,4-pentanediol (MPD) were purchased from Hampton Research. Native TBA and TBA mutants were purchased from Integrated DNA Technologies (IDT), Shanghai Generay company or synthesized and purified in house.

### Crystallization of TBA-Pb^2+^ complexes

Lead chloride, native TBA (5′-GGTTGGTGTGGTTGG-3′, Fig. 1A) and TBA mutants were all dissolved in ultra-pure water. Mix native TBA (or mutants) and lead chloride with a final concentration of 1.0 mM and 2.0 mM, respectively. Incubate the sample at room temperature for 30 min prior to crystallization. The initial crystallization conditions were identified using the Gryphon crystallization robot system from Art Robbins Instrument and crystallization kits from Hampton Research. The sitting-drop vapor diffusion method was used during the initial screening at 16 °C, whereas all the crystallization optimization procedure were performed at 18 °C using the hanging-drop vapor diffusion method.

Well solutions are composed of 30% v/v MPD for all the crystals. The drops contain 1.6 μl TBA-Pb^2+^ sample and 0.4 μl crystallization buffer (10% v/v MPD, 40 mM sodium cacodylate pH 7.0, 12 mM spermine tetra-HCl, and 80 mM sodium chloride) for the A-form crystals, whereas they are composed of equal volume (1.0 μl) of sample and crystallization buffer for the B-form crystals. The drops are composed of 0.7 μl sample and 0.7 μl crystallization buffer (10% v/v MPD, 40 mM sodium cacodylate pH 7.0, 28 mM spermine tetra-HCl, and 80 mM sodium chloride) for the G8C-Pb^2+^ complex crystals. Growth of the crystals are slow. They normally appeared 2 weeks after the crystallization and reached the full sizes in another 2 weeks.

### Diffraction data collection

All the crystals were cryoprotected using 30% v/v MPD and flash-frozen by quickly dipping into liquid nitrogen. The X-ray diffraction data were collected on beamline BL17U1, BL18U1, and BL19U1 at Shanghai Synchrotron Radiation Facility (SSRF, Shanghai, China) at cryogenic temperature, maintained with cryogenic system. One single crystal was used for each structure; data processing was carried out using the HKL2000 or HKL3000 programs^34^. The data collection and processing statistics were summarized in Table 1.

### Structure determination and refinement

The A-form TBA-Pb^2+^ complex structure was solved by the SAD (single anomalous diffraction) method^35^ using the anomalous signals of Pb^2+^. The Autosol program embedded in the phenix suite^36^ could identify two Pb^2+^ ions with figure-of-merit (FAM) value of 0.741. The resulting electron density maps were used as guide for manually model building by Coot program^37^. The model were then refined by the Refmac5 program embedded in the CCP4i suite^38^. 5% randomly selected data was set aside for free R-factor cross validation calculations during the refinement. Both the B-form TBA-Pb^2+^ complex structure and the G8C-Pb^2+^ complex structure were solved by the molecular replacement method using the phaser program^39^, the A-form structure was used as the search model. The final refinement of the three structures were all done using the phenix.refine program of phenix. The detailed refinement statistics were summarized in Table 1.

### Circular dichroism (CD) spectroscopy

All native and mutant TBAs (Table 3) utilized in the circular dichroism (CD) spectra analysis were synthesized by solid phase synthesizer and purified by High Performance Liquid Chromatography (HPLC) in the laboratory. All samples were prepared by mixing TBA (or mutants) and lead nitrate that were dissolved in 10 mM Tris acetate buffer (pH 7.2). The final TBA and lead nitrate concentrations are 10 μM and 50 μM, respectively. All solutions were heated to 85 °C for 3 min, then cooled slowly to room temperature and stored at 4 °C for overnight. CD studies were carried out in utilizing a Jasco-815 CD spectrometer in a quartz cell with a 10-mm path length. CD spectra were collected from 360 to 260 nm and with a scanning speed of 100 nm/min. The bandwidth was set to 1.0 nm, and the digital integration time was 1.0 s. All CD spectra were base line corrected against the blank buffer.

## Supplementary Information

Supplementary Figures.

## Data Availability

The atomic coordinates and structure factors have been deposited in the Protein Data Bank (PDB, www.pdb.org) under accession ID codes 7D31, 7D32, and 7D33 for the A-form TBA-Pb^2+^ complex, the B-form TBA-Pb^2+^ complex and the G8C-Pb^2+^ complex, respectively.
