# Supplementary material for: Structure-guided development of Pb2+-binding DNA aptamers
Source: Sci Rep. 2022 Jan 10;12:460. doi: 10.1038/s41598-021-04243-2 (PMC8748701; doi:10.1038/s41598-021-04243-2)
Supplement: Supplementary file 1 — Supplementary Figures. [file 41598_2021_4243_MOESM1_ESM.pdf]

Supporting Information

For

Structure-guided Development of Pb^{2+}-binding DNA Aptamers

Hehua Liu[1,+], Yanqing Gao[1,+], Johnsi Mathivanan[2,+], Fusheng Shen[2], Xi Chen[1], Yangyang Li[1], Zhiwei Shao[1], Yixi Zhang[1], Qiyuan Shao[1], Jia Sheng[2,*], Jianhua Gan[1,*]

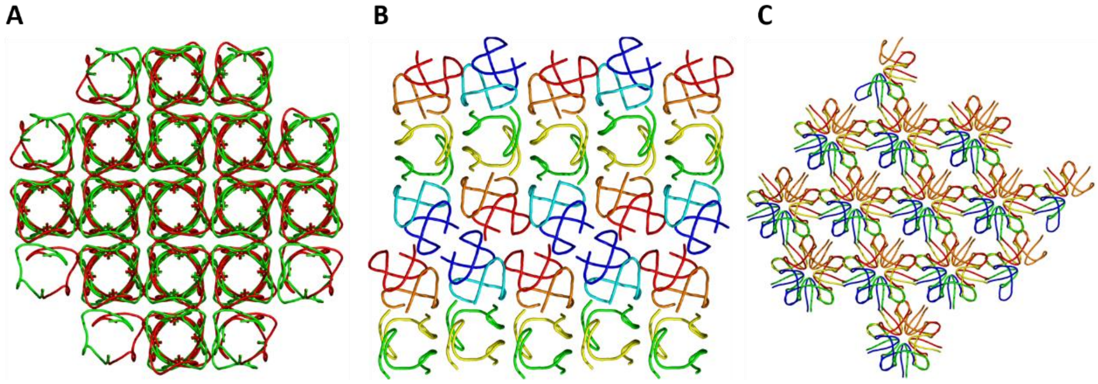

Fig. S1: Packing of (**A**) A-form TBA-Pb^{2+} complex, (**B**) B-form TBA-Pb^{2+} complex and (**C**) G8C-Pb^{2+} complex in the crystal lattice. Figures were displayed using PyMOL (http://www.pymol.org/).

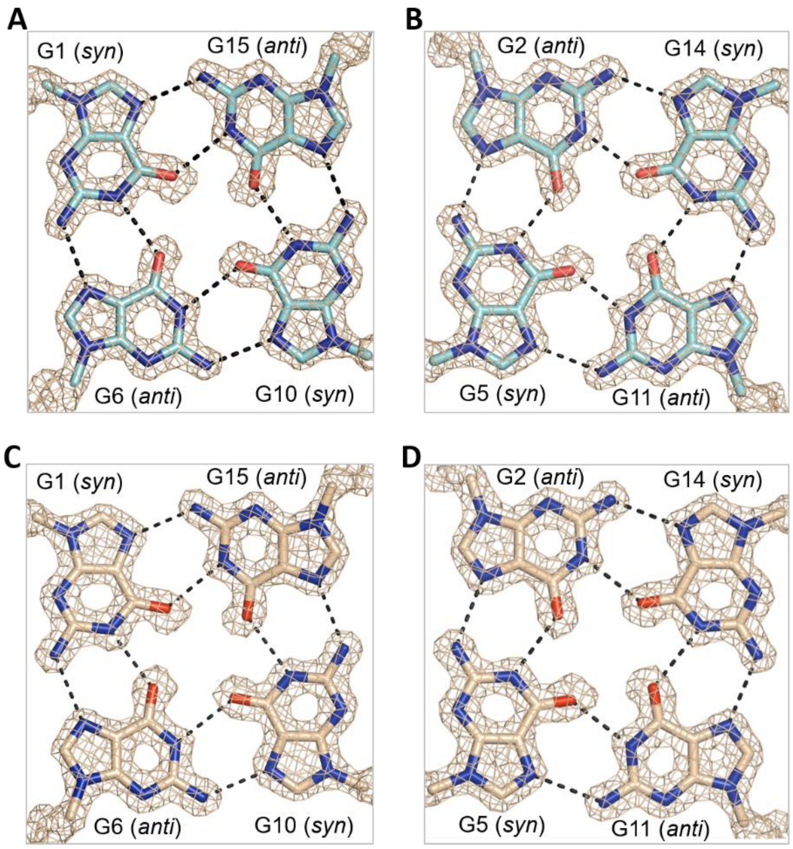

Fig. S2: G-tetrads observed in the A-form TBA-Pb^{2+} complex structure. (**A-B**) G-tetrads formed by TBA molecule A. (**C-D**) G-tetrads formed by TBA molecule B. Nucleobases of the G-tetrads are shown as sticks outlined with $2F_o$-F_c electron density maps (contoured at 1.5 σ level). The N1-O6 and N2-N7 H-bond interactions are indicated by black dashed lines. Figures were displayed using PyMOL (http://www.pymol.org/).

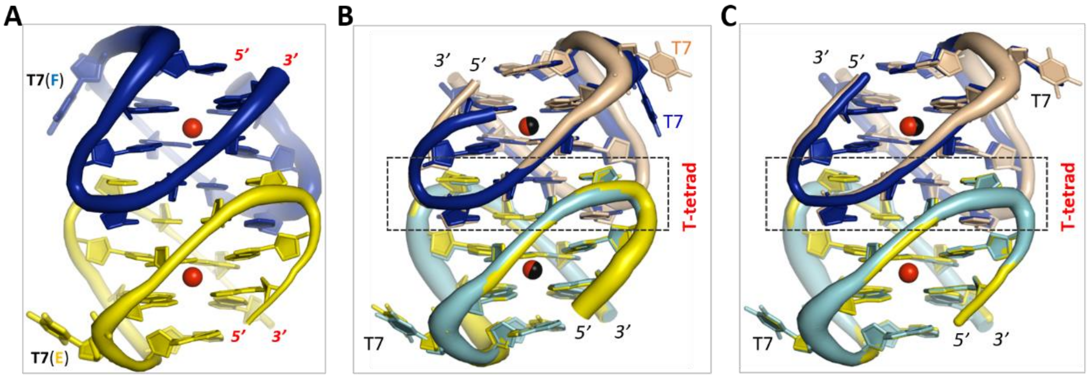

Fig. S3: Comparison of the A- and B-form TBA-Pb^{2+} structure. (A) Overall folding of the EF dimer of the B-form structure. (B) Superposition of the AB dimer of the B-form structure with the A-form structure. (C) Superposition of the CD dimer of the B-form structure with the A-form structure. All dimers of the B-form structure are colored in yellow and blue. The A-form structure is colored in cyan and wheat in panels (B) and (C). Figures were displayed using PyMOL (http://www.pymol.org/).

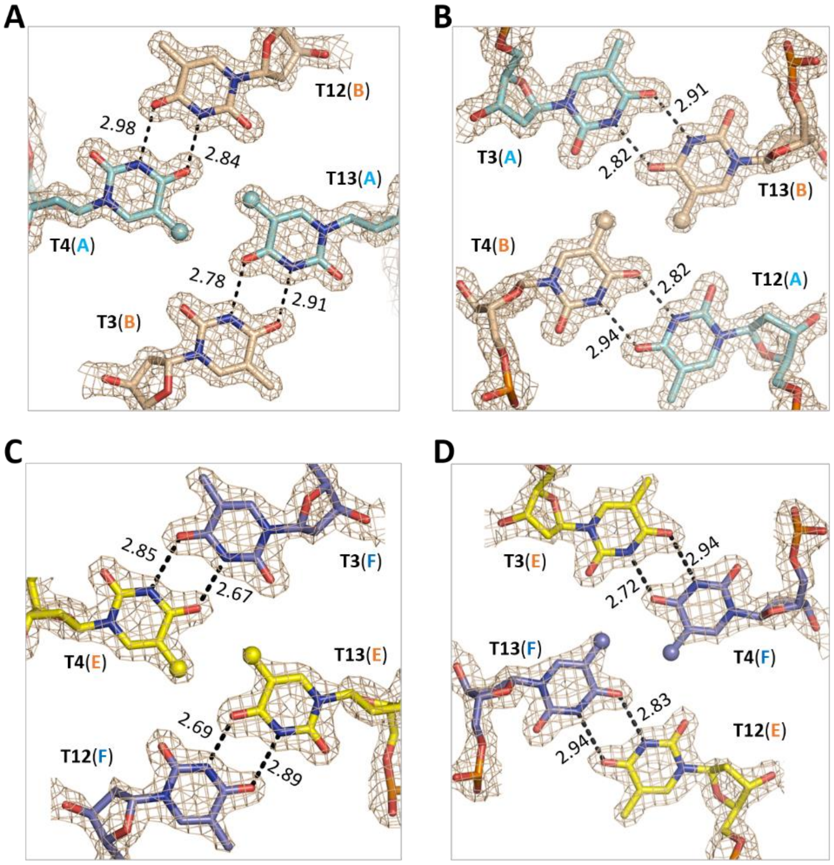

Fig. S4: Interactions formed between the TT linker nucleotides. (**A-B**) Detailed H-bond interactions formed between the T3T4 and T12T13 linkers of the A-form TBA-Pb^{2+} structure. (**C-D**) Detailed H-bond interactions formed between the T3T4 and T12T13 linkers of the EF dimer of the B-form TBA-Pb^{2+} structure. The 5-methyl groups of T4 and T13 are shown as spheres. The $2F_o$-F_c electron density maps are contoured at 1.5 σ level in panels (**A**) and (**B**), whereas they are contoured at 1.2 σ level in panels (**C**) and (**D**). Figures were displayed using PyMOL (http://www.pymol.org/).

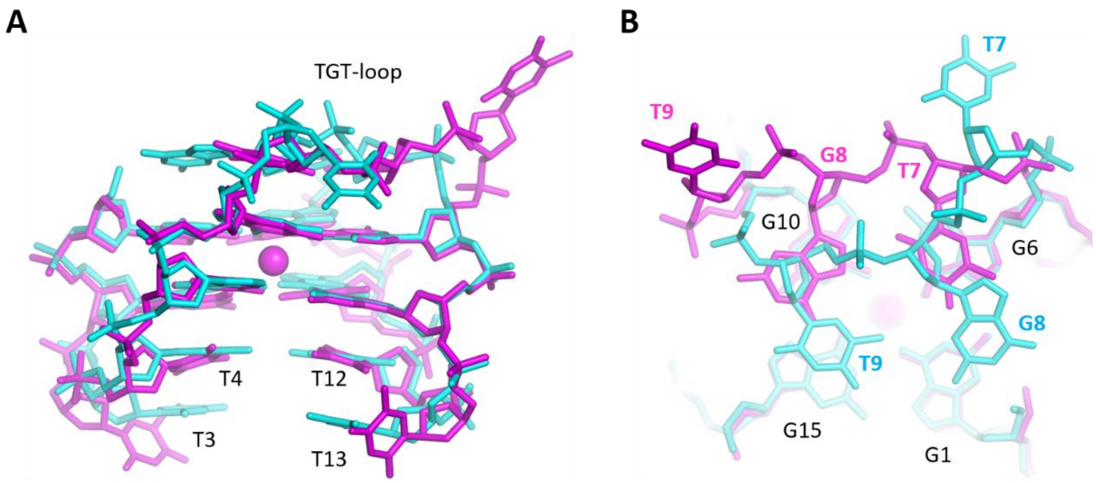

Fig. S5: (**A**) Superposition of TBA molecules of the A-form TBA-Pb^{2+} complex and the TBA-thrombin complex (PDB_ID: 4DII). (**B**) Superposition showing the conformational difference of T7G8T9 linkers. The Pb^{2+}-complexed TBA molecule is shown as sticks in cyan. TBA and K+ observed in the TBA-thrombin complex are shown as magenta sticks and sphere, respectively. Figures were displayed using PyMOL (http://www.pymol.org/).

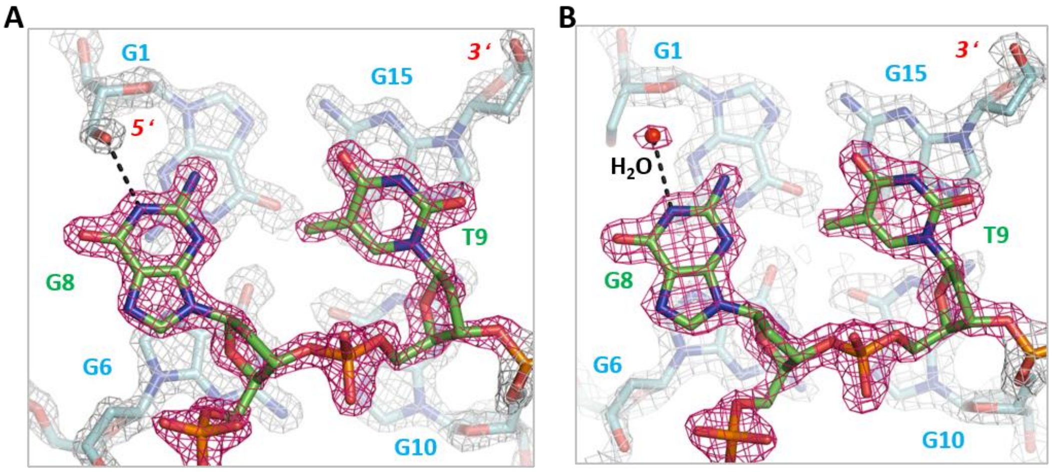

Fig. S6: Conformation and interaction of G8 in (**A**) the A-form and (**B**) the B-form complex structures. G8, T9 and nucleotides of the G-tetrad are shown as sticks. Water molecules are show as red spheres. The $2F_o$-F_c electron density maps are all contoured at 1.5 σ level.
